# Designing a Robust Kelvin Probe Setup Optimized for Long-Term Surface Photovoltage Acquisition

**DOI:** 10.3390/s18114068

**Published:** 2018-11-21

**Authors:** Elke Beyreuther, Stefan Grafström, Lukas M. Eng

**Affiliations:** 1 Institut für Angewandte Physik, Technische Universität Dresden, Nöthnitzer Str. 61, 01187 Dresden, Germany; lukas.eng@tu-dresden.de; 2 Fakultät Physik, Technische Universität Dresden, Haeckelstr. 3, 01069 Dresden, Germany; stefan.grafstroem@tu-dresden.de

**Keywords:** Kelvin probe, surface photovoltage, SPV, electronic defect states, surface states, contact potential difference, CPD, SrTiO_3_, wide-bandgap semiconductor, photorelaxation

## Abstract

We introduce a robust low-budget Kelvin probe design that is optimized for the long-term acquisition of surface photovoltage (SPV) data, especially developed for highly resistive systems, which exhibit—in contrast to conventional semiconductors—very slow photoinduced charge relaxation processes in the range of hours and days. The device provides convenient optical access to the sample, as well as high mechanical and electrical stability due to off-resonance operation, showing a noise band as narrow as 1 mV. Furthermore, the acquisition of temperature-dependent SPV transients necessary for SPV-based deep-level transient spectroscopy becomes easily possible. The performance of the instrument is demonstrated by recording long-term SPV transients of the ultra-slowly relaxing model oxide strontium titanate (SrTiO${}_{3}$) over 20 h.

## 1. Introduction

The electrical characterization of wide-bandgap materials, to which a variety of current topical material systems such as functional complex oxide structures [1] belongs, has been a methodological challenge for decades [2]. On the one hand, the electrical transport across these structures is crucially influenced by electronic defect states, especially at surfaces and interfaces [3]; we thus need reliable experimental techniques for analyzing the defect state distribution across a given bulk, film-substrate, hetero-, or device structure. On the other hand, wide-bandgap materials show low intrinsic carrier concentrations, making it difficult to apply conventional purely electrical characterization methods such as capacitance-voltage spectroscopy or deep-level transient spectroscopy. Optical methods are one way to overcome this problem; see, e.g., [2,4].

In this context, a promising and very versatile contactless technique to characterize the (surface- and interface-related) distribution of defect states in wide-bandgap materials and to extract their parameters is provided by the analysis of the surface photovoltage [5] as a function of:

- wavelength (known as surface photovoltage spectroscopy (SPS) [6,7,8]),
- light intensity,
- time (SPV transients), or
- temperature and time (SPV-based deep-level transient spectroscopy [9] (SPV-DLTS) [10,11]).

In brief, the SPV is defined to be the light-induced shift of the band bending of a semiconductor surface or interface. In principle, this shift can be detected by any method that is sensitive to the work function. As explained in [5], for highly resistive wide-bandgap samples with slow photorelaxation, the Kelvin probe detection of the SPV, which can be operated with continuous illumination, is the method of choice (while for monitoring materials showing fast SPV processes, a variety of other sophisticated detection schemes with high temporal resolution has been proposed; see, e.g., [12] and the references therein). Note that, apart from defect physics of semiconductors being in focus here, there are also other contexts, in which the SPV as measured by the Kelvin probe is employed as the analysis tool, for example in gas sensing devices (e.g., [13] and the references therein).

In general, the Kelvin probe measures the difference in work function between the sample surface under investigation and an adjacent reference electrode, which together form a capacitor [14]. When the sample and the reference electrode are electrically connected without any external bias, the difference in work function appears as a potential difference between these two surfaces, called the contact potential difference $U_{CPD}$. Hence, the surfaces become charged in accordance with their capacitance. The goal of the Kelvin probe is to find exactly that external bias voltage $U_{ext}$ (in the following also referred to as the Kelvin signal) that discharges the capacitor. This voltage equals $-U_{CPD}$. To find the discharged state, one modulates the capacitance by oscillating the reference electrode perpendicularly to the sample surface. A control loop then feeds back a bias voltage such that the alternating current induced by the vibration is nullified (see, e.g., [15,16,17,18,19,20,21,22,23,24,25,26] for various designs presented over the years).

To get the surface photovoltage, we have to measure the difference between the Kelvin signals under illumination and in the dark: $SPV:=U_{CPD}^{illum}-U_{CPD}^{dark}$. Due to the differential nature of such a measurement, the exact work function of the reference electrode need not be known.

In the present paper, we describe (after some fundamental considerations on the Kelvin probe current, from which important experimental boundary conditions can be derived) a low-cost Kelvin probe, which provides—in contrast to commercial needle-/tip-based designs, being vulnerable to inhomogeneous sample illumination due to shadowing—full and easy optical access to the sample, as well as mechanical and electrical long-term stability and is thus able to acquire reliable SPV transients over hours and days. The performance of the setup is demonstrated by means of SPV transients of a SrTiO${}_{3}$ single crystal.

## 2. Theoretical Considerations

**Figure 1:**
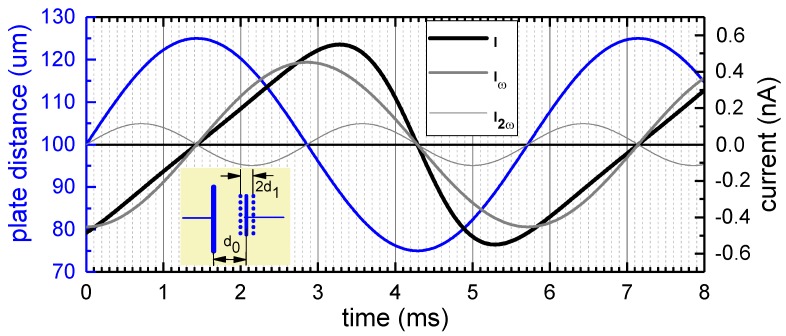
Comparative plot of the capacitor plate oscillation $\frac{d_{1}}{d_{0}}\sin\left(\omega t\right)$ (see the configuration sketched in the inset and blue curve), the induced alternating current according to Equation (2) (black curve), as well as its first two harmonics (gray curves, cf. Equations (5) and (6), and see also Figure 2) with numerical values taken from the real experimental setup described in Section 3: $A=\frac{\pi }{4}\left(5\times 10^{-3}\right)$${}^{2}$m${}^{2}$; U=1 V; ω=2·π·175 Hz; $d_{0}=100$ μm; $d_{1}=25$ μm.

We start with the mathematical description of the alternating current and its first two harmonics within a parallel-plate capacitor setup, where one plate oscillates perpendicularly to the other one. This represents a classical Kelvin probe setup. The capacitance $C=\epsilon \epsilon_{0}\frac{A}{d}$, with $\epsilon_{0}$ being the vacuum permittivity, ϵ the dielectric constant of the material filling the gap between the capacitor plates (since ϵ=1 for an air gap, we omit ϵ in the following), *A* the plate area, and *d* the plate distance. Whenever *d* is modulated in a sinusoidal way, i.e., $d=d_{0}+d_{1}\sin\left(\omega t\right)$, at an angular frequency ω, with $d_{0}$ being the mean plate distance and $d_{1}$ the maximum oscillation elongation, the charge *Q* follows as:

(1) $$Q=C\cdot U=\epsilon_{0}\frac{AU}{d_{0}+d_{1}\sin\left(\omega t\right)},$$

where $U=U_{ext}+U_{CPD}$ is the voltage between the plates. The oscillation of *d* gives rise to an alternating current (see Figure 1):

(2) $$I\left(t\right)=\frac{dQ}{dt}=-\frac{\epsilon_{0}AUd_{1}\omega \cos\left(\omega t\right)}{{\left[d_{0}+d_{1}\sin\left(\omega t\right)\right]}^{2}}.$$

After a suitable power series expansion in terms of $\frac{d_{1}}{d_{0}}\sin\left(\omega t\right)$ followed by algebraic transformations (see the Supplementary Materials), the *k*th harmonic of the current *I* is found to be:

(3) $$I_{k\omega }=kb\frac{i^{k-1}}{2}\left\{e^{ik\omega t}+{\left(-1\right)}^{k-1}e^{-ik\omega t}\right\}\sum_{n=0}^{\infty }{\left(\frac{a}{2}\right)}^{2n+k-1}\frac{(2n+k)!}{n!(n+k)!}\;,$$

where:

(4) $$a:=\frac{d_{1}}{d_{0}}\;;\;b:=-\epsilon_{0}AU\omega \frac{d_{1}}{d_{0}^{2}}\;.$$

The first and second harmonic part of *I* read as follows:

(5) $$\begin{array}{ccc} I_{\omega } & = & -\epsilon_{0}AU\omega \frac{d_{1}}{d_{0}^{2}}\cos\left(\omega t\right)\sum_{n=0}^{\infty }{\left(\frac{1}{2}\frac{d_{1}}{d_{0}}\right)}^{2n}\frac{(2n+1)!}{n!(n+1)!} \end{array}$$

(6) $$\begin{array}{ccc} I_{2\omega } & = & 2\epsilon_{0}AU\omega \frac{d_{1}}{d_{0}^{2}}\sin\left(2\omega t\right)\sum_{n=0}^{\infty }{\left(\frac{1}{2}\frac{d_{1}}{d_{0}}\right)}^{2n+1}\frac{(2n+2)!}{n!(n+2)!}\;. \end{array}$$

**Figure 2:**
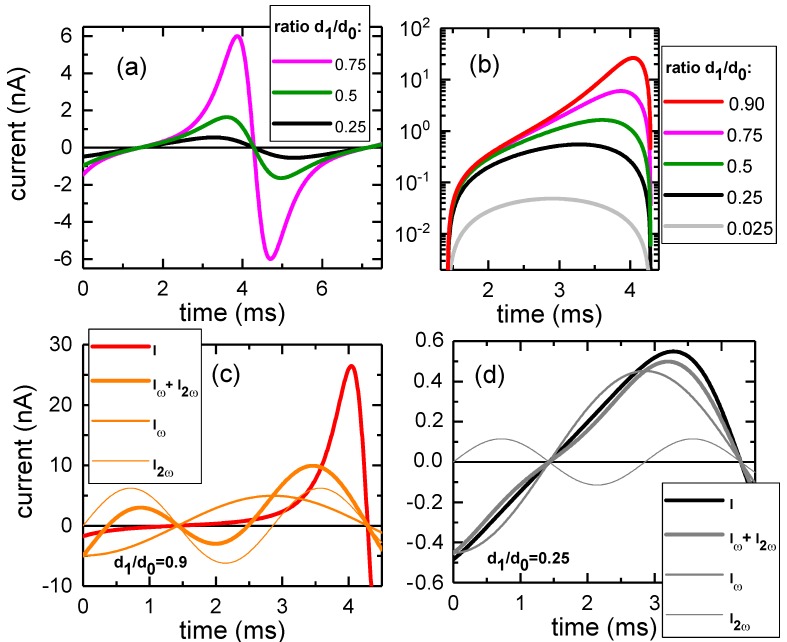
(**a**) Plot of the Kelvin probe current *I* as given in Equation (2) for three different ratios $d_{1}/d_{0}$ with numerical values given already in Figure 1. (**b**) Logarithmic plot of the region of the amplitude maximum of *I* showing the decisive influence of the ratio $d_{1}/d_{0}$ on both the maximum induced current (which can vary over several orders of magnitude) and its asymmetry. Panels (**c**) and (**d**) illustrate the decomposition of *I* into harmonics: while for a comparatively large $d_{1}/d_{0}$ value, as depicted in (**c**), more than two harmonics would be needed to rebuild the current, for moderate values as shown in (**d**)—which corresponds approximately to our real experimental conditions—already the first harmonic represents the current well.

As seen from Equation (2), the current vanishes at the turning points of the mechanical vibration of the capacitor plate (Figure 1). For $d_{1}/d_{0}\ll 1$, I(t) is well described by the first harmonic $I_{\omega }$, Equation (5). When $d_{1}$ comes closer to $d_{0}$, I(t) becomes strongly asymmetric with respect to the points of zero displacement, with positive and negative current peaks appearing close to the inner turning point. This corresponds to the higher harmonics becoming obviously more prominent (see Figure 2c,d in comparison). At $d_{1}/d_{0}>0.8$ (see the Supplementary Materials for details), the second harmonic is indeed higher in amplitude than the first harmonic. Under real experimental conditions, it appears practical to choose $d_{1}/d_{0}$ less than 0.5 in order to avoid physical probe-sample contact safely. For that case, the first harmonic still clearly dominates, so that operating the feedback loop with the first harmonic gives the highest sensitivity.

## 3. Design Description

### 3.1. Mechanical Construction

**Figure 3:**
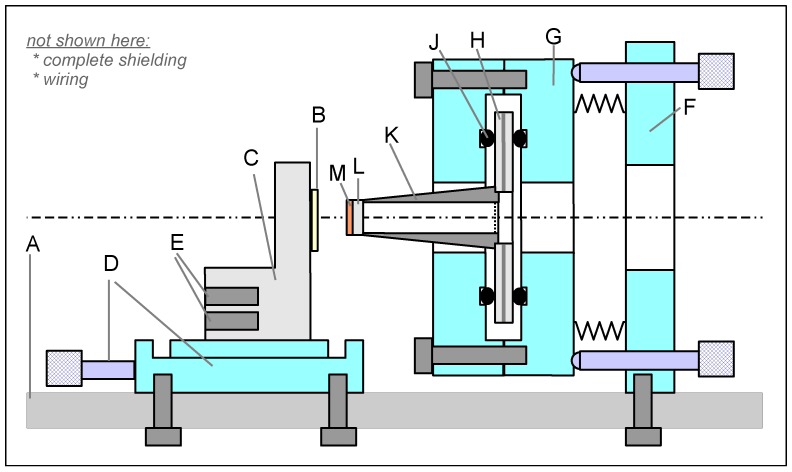
Mechanical parts: (A) base plate; (B) sample fixed on a (C) block with several (E) bores for optional heating cartridges and temperature sensors; (D) translation stage for sample movement; Parts (F) and (G) represent the probe mount, with (F) being fixed and (G) tiltable around two axes; (H) piezoelectric bimorph clamped between (J) rubber rings within the metallic housing (G) for efficient decoupling of the piezo excitation signal from the probe current; (K) hollow cylinder glued to the piezo, carrying the (L) quartz window with the (M) metal film that serves as the probe. Note that the sketch, which is mainly a cross-section, is not to scale. In a true cross-section, only one of the two probe adjustment screws would be visible, since they are placed at diagonal corners of part (F). The whole construction is surrounded by a grounded metal box (not shown), which serves for both electrical shielding and stray light protection. An entrance slit provides optical access. For photographs of the device, refer to Figures S2 and S3 in the Supplementary Materials.

The mechanical construction (Figure 3) is placed in a grounded metal (aluminum) box, which provides both electrical shielding and stray light protection. Note that parts not explicitly specified by a supplier and a model number are home-built. The box has an entrance slit for coupling light from the illumination setup to the sample surface. The illumination setup can vary and is based either on a laser source or a monochromatized white-light source. In both cases, the photon flux is kept at a certain set point value by a feedback loop controlling the position of a continuous neutral-density filter placed on a motorized translation table. The probe and sample holders are attached to the base plate of the box. To enhance the mechanical stability, the Kelvin probe box is—together with the illumination setup—mounted on a vibration-isolating table.

The sample holder consists of two parts: (i) a linear translation stage (D) for sample approach towards the probe and (ii) a copper block (C) to which the sample (B) is glued by conducting silver paste. For temperature-dependent SPV measurements, especially SPV-DLTS (deep-level transient spectroscopy) [10,11], the copper block includes bores (E) containing heating cartridges and a Pt-100 temperature sensor. To prevent the system from accidental overheating during overnight data acquisition, a temperature-sensitive fuse glued on the copper block is included in the heating circuit.

The probe holder is designed as follows: An annular piezoelectric bimorph element (H; CBM 100/50–10/120 M by Piezomechanik GmbH Munich, Germany; maximal center displacement: 120 μm at an applied voltage of 100 V, resonance frequency: 5 kHz, outer diameter: 50 mm, inner diameter: 10 mm, thickness: 0.6 mm) is clamped between rubber O-rings (J) inside a metal housing. The bimorph actually consists of two piezoelectric disks glued onto each other with an electrode in between. The driving voltage is applied to this inner electrode, while the electrodes on the outer surfaces are grounded. This arrangement provides an excellent shielding and prevents cross-coupling of the piezo excitation voltage to the probe-sample circuit. The piezo disk carries an aluminum hollow cylinder (K), to which the probe (L,M) is attached. The outer diameter of the cylinder is slightly tapered.

The probe (L) consists of a quartz window (Model 45463, by Edmund Optics) measuring 5 mm in diameter and 1 mm in thickness, having a semitransparent metal layer (M) evaporated onto it, and is glued to the narrow end of the cylinder. The metal layer consists of a 2-nm-thick chromium adhesion layer and an 8-nm-thick gold film, which serves as the inert reference electrode. The sample thus can be illuminated through the probe without shadowing effects. The probe has a much larger area than in most other Kelvin probe designs, which use a needle as the reference electrode. The large area gives a large current amplitude and is hence favorable in terms of signal-to-noise ratio. The device measures integral CPD or SPV values averaged over the probe area.

Special care was taken concerning the adjustability of the probe: the whole unit (F,G) carrying the piezo and the probe can be tilted around two orthogonal axes by micrometer screws to achieve a parallel alignment of the surfaces of the probe and the sample. For the alignment procedure, a He-Ne laser beam hits the sample surface at near-normal incidence. The back reflections from both the probe and the sample are monitored on a screen at a large distance. As long as the two surfaces are not parallel, several reflected spots are visible. By adjusting the two micrometer screws of the probe mount, the spots can be superimposed on each other and consequently be made to interfere. As soon as interference is observed, one can be certain that the probe and the sample are aligned parallel. Once adjusted parallel to the probe surface, the sample can be approached by a micrometer screw until the Kelvin signal settles to a distance-independent value.

The probe-to-sample spacing measures ∼100 μm, whereas the oscillation amplitude is set to 25 μm. For the piezoelectric bimorph used here, this displacement can be realized by a relatively low excitation voltage of approximately 20 V, which is favorable in terms of as little disturbance of the current measurement as possible. Another decisive advantage of the large elongation of the piezoelectric bimorph is that it can be operated off resonance. Resonant operation typically suffers from long-term stability problems, which need further sophisticated electronic circuitry to be overcome [17,26]. In our case, the vibration is stable over several days in amplitude and phase.

### 3.2. Electrical Circuitry

**Figure 4:**
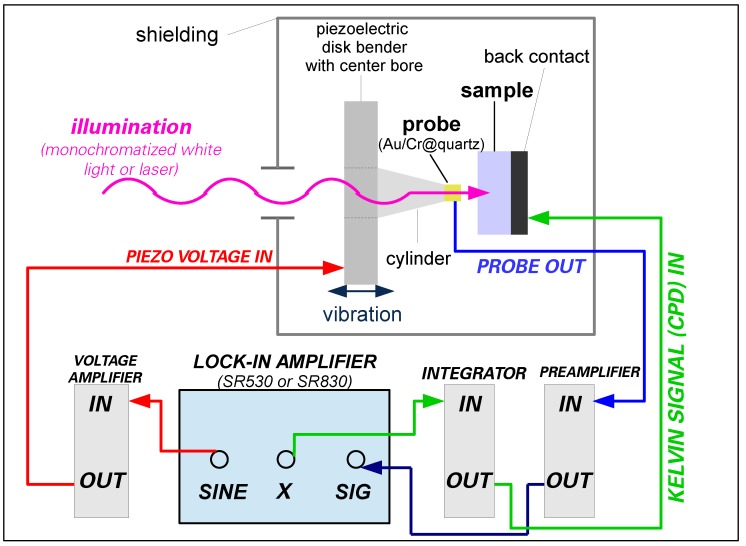
Electrical circuitry: The piezoelectric disk bender is excited by a sinusoidal voltage, while the first harmonic of the preamplified probe current is analyzed by a lock-in amplifier. The in-phase part of this first harmonic serves as the input signal for the integrating controller, whose output is fed back to the sample rear contact in order to nullify the probe current. This voltage then corresponds to the contact potential difference between the probe and sample surface. For SPV measurements, light from an illumination setup is coupled through the probe onto the sample. Note that the wiring for the piezo voltage is led through the box at the opposite site of the other signals to prevent crosstalk.

Figure 4 shows the electrical signal flow: For piezo excitation, the internal sine generator of a lock-in amplifier (Stanford Research Systems, Model 530 or 830) delivers a voltage (“SINE”) with an rms amplitude of 2 V and a frequency of 175 Hz, which is amplified by a home-built voltage amplifier (gain = 10) and fed to the inner contact of the piezo disk. The current *I* generated in the vibrating capacitor structure is fed from the sample rear contact via a shielded cable into a home-built current-to-voltage converter (preamplifier) with a transimpedance of 1 MΩ. The preamplifier is located close to the Kelvin probe box to keep the cable capacitance as low as possible. The output voltage of the preamplifier acts as the input to the lock-in amplifier (LIA), which extracts the in-phase signal amplitude (“X”) of the first harmonic (see Section 2 and the Supplementary Materials for a more detailed mathematical discussion of the Kelvin probe current and its harmonics). The LIA is typically operated with a sensitivity between 20 and 100 mV and a time constant of 100 ms. The phase offset of the LIA is chosen such that the full signal appears at the “X” output. This “X signal” is fed back to the probe via a home-built integrator (“I controller”) delivering the external bias voltage that nullifies the current *I*. The integrator output voltage then equals $-U_{CPD}$. After removal of high-frequency noise by a low-pass filter and subsequent analog-to-digital conversion by a multifunction data acquisition board (Model PCI-MIO-16XE-10, National Instruments), this voltage is recorded by the controlling computer. Not only the recording of the “Kelvin signal” $U_{CPD}$, but also all further data logging and instrument control software, e.g., for wavelength selection, photon flux, and shutter control, as well as optional temperature control for the long-term experiments (adjustment of the heating current by a (software) PID loop) were developed on the basis of National Instruments LabVIEW.

## 4. Results and Discussion

**Figure 5:**
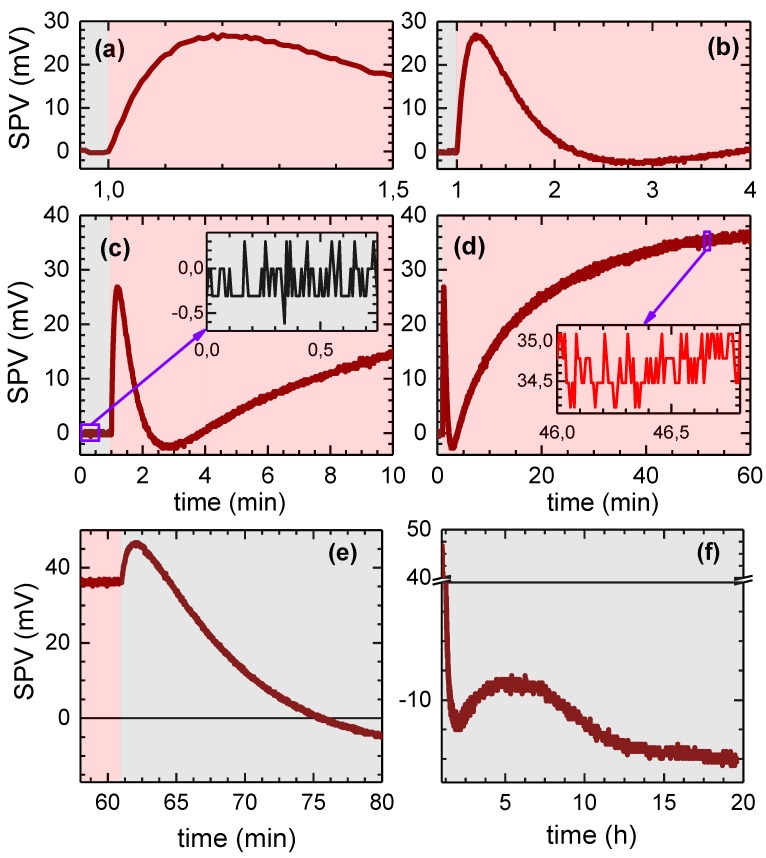
(**a**) Temporal development of the SPV of a SrTiO${}_{3}$ single crystal under on- and off-switching of 600-nm illumination (photon flux: ∼10${}^{13}\;$s${}^{-1}$; intensity: 20 μW illuminating the whole area under the 5-mm-diameter probe, corresponding to 0.1 mW/cm${}^{2}$, a regime where heating effects can be neglected; see the estimation in [33]) with grey regions indicating darkness periods and light red regions symbolizing illumination periods, respectively. Diagrams (**a**–**d**) show detailed plots of the “light-on” photoresponse, zooming into different time regimes, and reveal the simultaneous presence of several carrier exchange processes with different time constants. The insets in (**c**) and (**d**) demonstrate noise bands smaller than 1 mV for both the dark and the illuminated case, being at the resolution limit of the 16-bit A/D converter. Diagrams (**e**) and (**f**) visualize the SPV’s relaxation behavior after switching off the illumination. Note that multiple carrier exchange processes are visible, as well, and no recovery to the initial value of $U_{CPD}^{dark}$, which would correspond to reaching the zero line in the above SPV diagrams, can be observed within the given 20 h.

Figure 5 demonstrates the performance of the Kelvin probe by means of SPV transients of a highly resistive SrTiO${}_{3}$ single crystal (Crystec GmbH, Berlin, Germany), which represents a prototypical wide-bandgap material exhibiting very slow photorelaxation—while some reference data acquired on n- and p-doped silicon (showing rapid photorelaxation), whose SPV response is well described in the literature, are shown and briefly discussed in the Supplementary Materials (Figure S4). The curves in Diagrams (a)–(d) depict the temporal development of the SPV under (exemplary) 600-nm (= sub-bandgap) illumination from a monochromatized white-light source with stabilized light intensity. Note that for the first minute of data acquisition, the sample was kept in the dark to gain a value for the actual darkness contact potential difference $U_{CPD}^{dark}$. To make the obvious existence of multiple charge transfer processes with different time constants visible, four different time frames were chosen. The insets of Diagrams (c) and (d) show the noise bands in the dark and under illumination to be smaller than 1 mV, which is the state-of-the-art for SPV measurements using unmodulated illumination schemes. The subsequent relaxation in the dark can be seen in Diagrams (e) and (f) and was recorded over a time span of about 20 h. In a typical full SPV analysis, comparative datasets of such light-on and light-off transients would be collected for a number of relevant wavelengths under varied light intensity and/or temperature to be processed using one or more mathematical models (see, e.g., [27,28,29,30], or for a comparative summary, [31]) to extract a number of material parameters such as capture cross-sections for photons and electrons, activation energies of trap states, or surface/interface band bendings in the dark and under illumination. Prior to the time-consuming acquisition of transients, surface photovoltage spectra, i.e., the SPV as a function of wavelength, are usually recorded in order to select meaningful wavelengths for the recording of transients.

In principle, the setup can easily be adopted to measure the SPV capacitively with chopped illumination. For that, the probe would be statically brought close to the sample contact, illuminated with modulation, and the induced alternating current, which is proportional to the SPV, analyzed with regard to its amplitude and phase; see e.g., [32] and references therein. However, this method would be limited to systems exhibiting rapid photorelaxation.

## 5. Conclusions

In summary, we have explained the design and performance of a Kelvin probe setup optimized for monitoring ultra-slow surface photovoltage relaxation processes. The apparatus provides (i) long-term stability due to off-resonance piezo operation, (ii) a high signal-to-noise ratio because of a large probe area, low-voltage piezo excitation, and excellent electrical shielding, (iii) direct sample illumination through the probe, (iv) an SPV resolution of ∼1 mV, and (v) the opportunity to perform sample heating.

## Supplementary Materials

The following are available online at http://www.mdpi.com/1424-8220/18/11/4068/s1: pdf document with further mathematical details, photographs of the Kelvin probe device, and reference SPV data on n- and p-type silicon.
