# Supplementary material for: Designing a Robust Kelvin Probe Setup Optimized for Long-Term Surface Photovoltage Acquisition
Source: Sensors (Basel). 2018 Nov 21;18(11):4068. doi: 10.3390/s18114068 (PMC6264026; doi:10.3390/s18114068)
Supplement: Supplementary file 1 [file sensors-18-04068-s001.zip › sensors-381689-supplementary.pdf]

Supplementary Materials: Designing a Robust Kelvin Probe Setup Optimized for Long-Term Surface Photovoltage Acquisition

Elke Beyreuther, Stefan Grafström, and Lukas M. Eng

1. Decomposition of the Kelvin probe current into its harmonics

In order to obtain the amplitude of the kth harmonic of the current I (cf. eq. (5) of the main article):

$$I = -\frac{\epsilon\epsilon_0 A U d_1 \omega \cos(\omega t)}{[d_0 + d_1 \sin(\omega t)]^2} \quad . \tag{1}$$

we carry out a series expansion of equation (1), using the following abbreviations:

$$a := \frac{d_1}{d_0} \quad ; \quad b := -\epsilon\epsilon_0 A U \omega \frac{d_1}{d_0^2} \quad ; \quad x := \omega t \quad , \tag{2}$$

which simplifies equation (1) to:

$$I = b \cos x (1 + a \sin x)^{-2} \quad . \tag{3}$$

We use the following power series expansion, which follows from the binomial theorem [1]:

$$(1+z)^{-2} = 1 - 2z + 3z^2 - 4z^3 + 5z^4 - \ldots = \sum_{n=0}^{\infty} (-1)^n (n+1) z^n \quad \text{for} \quad |z| < 1 \quad . \tag{4}$$

In the present case, with $z = a \sin x$, the condition $|z| < 1$ is always fulfilled, because due to physical reasons the maximum oscillation amplitude d_1 of the vibrating capacitor plate must be smaller than the average distance d_0 between the two plates, which means $a := \frac{d_1}{d_0} < 1$. Taking relation (4) into account we can rewrite the expression for I:

$$I = \sum_{n=0}^{\infty} b(-a)^n (n+1) \cos x \sin^n x \quad . \tag{5}$$

After converting the sum (5) into expressions *without* powers of $\sin x$ using the Mathematica® command *TrigReduce* up to $n = 10$, we rearrange the respective result in order to find the coefficients for the harmonics $\cos x$, $\sin 2x$, $\cos 3x$, $\sin 4x$, $\cos 5x$, $\sin 6x$, aiming at finding a regular pattern that can be expanded to infinity. It turns out that the coefficients can be factorized as follows:

$$\begin{aligned}
&\cos x : + ba^0 (1 \cdot \frac{1}{1} \cdot \mathbf{1} \cdot a^0 + 3 \cdot \frac{1}{4} \cdot \mathbf{1} \cdot a^2 + 5 \cdot \frac{1}{16} \cdot \mathbf{2} \cdot a^4 + 7 \cdot \frac{1}{64} \cdot \mathbf{5} \cdot a^6 + 9 \cdot \frac{1}{256} \cdot \mathbf{14} \cdot a^8 + 11 \cdot \frac{1}{1024} \cdot \mathbf{42} \cdot a^{10} + \ldots) \\
&\sin 2x : - ba^1 (2 \cdot \frac{1}{2} \cdot \mathbf{1} \cdot a^0 + 4 \cdot \frac{1}{8} \cdot \mathbf{2} \cdot a^2 + 6 \cdot \frac{1}{32} \cdot \mathbf{5} \cdot a^4 + 8 \cdot \frac{1}{128} \cdot \mathbf{14} \cdot a^6 + 10 \cdot \frac{1}{512} \cdot \mathbf{42} \cdot a^8 + \ldots) \\
&\cos 3x : - ba^2 (3 \cdot \frac{1}{4} \cdot \mathbf{1} \cdot a^0 + 5 \cdot \frac{1}{16} \cdot \mathbf{3} \cdot a^2 + 7 \cdot \frac{1}{64} \cdot \mathbf{9} \cdot a^4 + 9 \cdot \frac{1}{256} \cdot \mathbf{28} \cdot a^6 + 11 \cdot \frac{1}{1024} \cdot \mathbf{90} \cdot a^8 + \ldots) \\
&\sin 4x : + ba^3 (4 \cdot \frac{1}{8} \cdot \mathbf{1} \cdot a^0 + 6 \cdot \frac{1}{32} \cdot \mathbf{4} \cdot a^2 + 8 \cdot \frac{1}{128} \cdot \mathbf{14} \cdot a^4 + 10 \cdot \frac{1}{512} \cdot \mathbf{48} \cdot a^6 + \ldots) \\
&\cos 5x : + ba^4 (5 \cdot \frac{1}{16} \cdot \mathbf{1} \cdot a^0 + 7 \cdot \frac{1}{64} \cdot \mathbf{5} \cdot a^2 + 9 \cdot \frac{1}{256} \cdot \mathbf{20} \cdot a^4 + 11 \cdot \frac{1}{1024} \cdot \mathbf{75} \cdot a^6 + \ldots) \\
&\sin 6x : - ba^5 (6 \cdot \frac{1}{32} \cdot \mathbf{1} \cdot a^0 + 8 \cdot \frac{1}{128} \cdot \mathbf{6} \cdot a^2 + 10 \cdot \frac{1}{512} \cdot \mathbf{27} \cdot a^4 + \ldots)
\end{aligned} \tag{6}$$

The terms in brackets obviously contain series of even powers of a, where the coefficients of each such power of a contain three factors. While the 1st and 2nd factors follow easy-to-find patterns, the third (bold printed) factors seem to be strange at first glance.

Actually, the numbers follow the rule of the CATALAN triangle[1] [2,3], in which the number C_{lm} at position (l, m) is given by

$$C_{lm} = \frac{(l+m)!(l-m+1)}{m!(l+1)!} \quad . \tag{7}$$

Here, the coefficient of $\cos x$ contains $C_{10}, C_{21}, C_{32}, \ldots$, for the coefficient of $\sin 2x$ the numbers $C_{20}, C_{31}, C_{42}, \ldots$ are relevant, and so forth. After several rearrangements a compact equation for the kth harmonic $I_{k\omega}$ of the KELVIN probe current I can be derived as:

$$I_{k\omega} = kb \sum_{n=0}^{\infty} \left(\frac{a}{2}\right)^{2n+k-1} \frac{(2n+k)!}{n!(n+k)!} \{(-1)^{(k-1)/2}\Theta[(-1)^{(k+1)}]\cos(kx) + (-1)^{k/2}\Theta[(-1)^{k}]\sin(kx)\} \quad , \tag{8}$$

with Θ being the HEAVISIDE function, which is needed because the harmonics alternately follow a sine or cosine function. Moreover, the terms $(-1)^{(k-1)/2}$ and $(-1)^{k/2}$ in the $\{\ldots\}$-term of equation (8) create the proper sign.

In order to remove the discontinuous HEAVISIDE function we use $(-1)^{1/2} = i$, as well as the EULER relations

$$\cos z = \frac{e^{iz} + e^{-iz}}{2} \quad ; \quad \sin z = \frac{e^{iz} - e^{-iz}}{2i} \quad , \tag{9}$$

and the relations

$$\Theta[(-1)^{(k+1)}] + \Theta[(-1)^{k}] = 1 \quad ; \quad \Theta[(-1)^{(k+1)}] - \Theta[(-1)^{k}] = (-1)^{k-1} \quad , \tag{10}$$

finally obtaining an alternative, more compact expression for $I_{k\omega}$:

$$I_{k\omega} = kb \sum_{n=0}^{\infty} \left(\frac{a}{2}\right)^{2n+k-1} \frac{(2n+k)!}{n!(n+k)!} \frac{i^{k-1}}{2} \{e^{ikx} + (-1)^{k-1}e^{-ikx}\} \quad . \tag{11}$$

The formulas (8) and (11) have been cross-checked with Mathematica® for $k = 1, 2, \ldots, 6$ (and n up to 10) to see whether they reproduce the coefficients from the expressions (6). The total current I can then be written as

$$I = \sum_{k=1}^{\infty} I_{k\omega} \quad . \tag{12}$$

The expressions for the first- and second-harmonic part of I turn out to be:

$$I_{\omega} = -\epsilon\epsilon_0 AU\omega \frac{d_1}{d_0^2} \cos(\omega t) \sum_{n=0}^{\infty} \left(\frac{1}{2}\frac{d_1}{d_0}\right)^{2n} \frac{(2n+1)!}{n!(n+1)!} \tag{13}$$

$$I_{2\omega} = 2\epsilon\epsilon_0 AU\omega \frac{d_1}{d_0^2} \sin(2\omega t) \sum_{n=0}^{\infty} \left(\frac{1}{2}\frac{d_1}{d_0}\right)^{2n+1} \frac{(2n+2)!}{n!(n+2)!} \quad , \tag{14}$$

where a, b and x have been substituted according to equations (2).

---

1 The numbers of the CATALAN triangle appear in many problems in combinatorics, but their appearance as part of the harmonics of the current within an oscillating paralel-plate capacitor is quite a surprise.

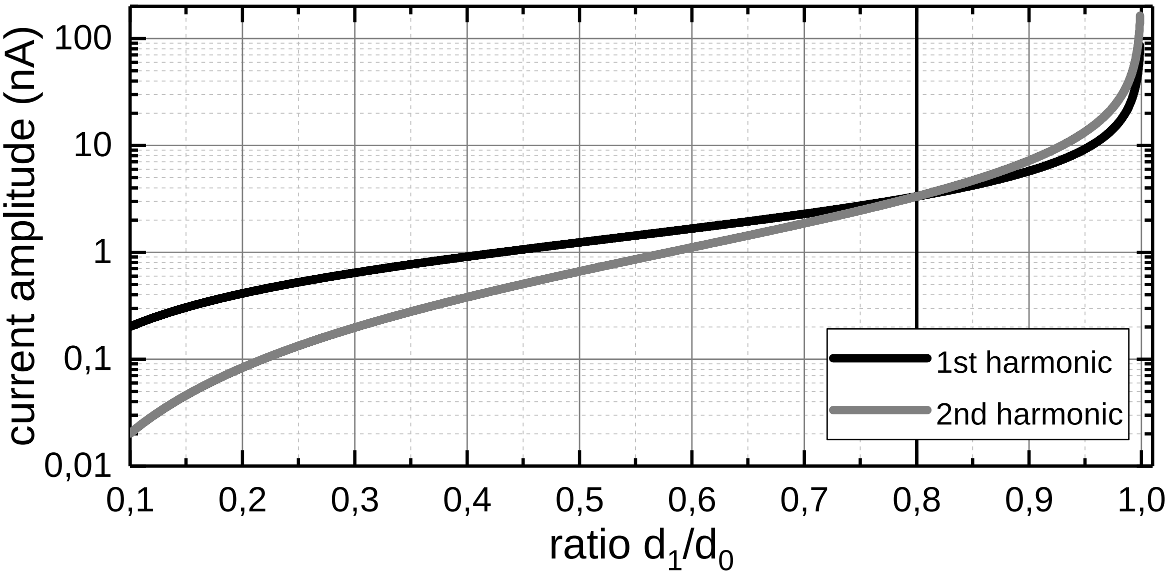

Figure S1. Kelvin-probe current amplitudes $\hat{I}_\omega$ and $\hat{I}_{2\omega}$ as a function of the ratio $a = \frac{d_1}{d_2}$ plotted with the parameters of the experiment described in the main article ($A = \frac{\pi}{4}(5 \cdot 10^{-3})\mathrm{m}^2$; $U = 1$ V; $\omega = 2 \cdot \pi \cdot 175$ Hz; $d_0 = 100\ \mu$m; $d_1 = 25\ \mu$m).

After a number of algebraic transformations, we find that the amplitude $\hat{I}_{k\omega}$ of the current according to eq. (11) is described by the hypergeometric function [4] ${}_2F_1$:

$$\hat{I}_{k\omega} = 2^{1-k} a^k k I_0 \quad {}_2F_1\left(\frac{1+k}{2}, \frac{2+k}{2}; 1+k; a^2\right) \tag{15}$$

with $I_0 = \frac{b}{a}$. Figure S1 depicts $\hat{I}_\omega$ and $\hat{I}_{2\omega}$ and their intersection point $\frac{d_1}{d_0} = 0.8$, which is independent of all the other variables.

2. Photographs of the Kelvin probe device

In order to illustrate the Kelvin probe device a bit more vividly, the following figures show some photographs (with partially removed outer shielding box). Figure S2 contains the first version in (a) side view and (b) top view, which corresponds to the construction described in the main article, while figure S3 shows a slightly modified version, not primarily devoted to oxide samples. There, the heating option is omitted, while the sample holder has been adapted to the sample-holder design of our UHV apparatus for preparing organic/inorganic interfaces.

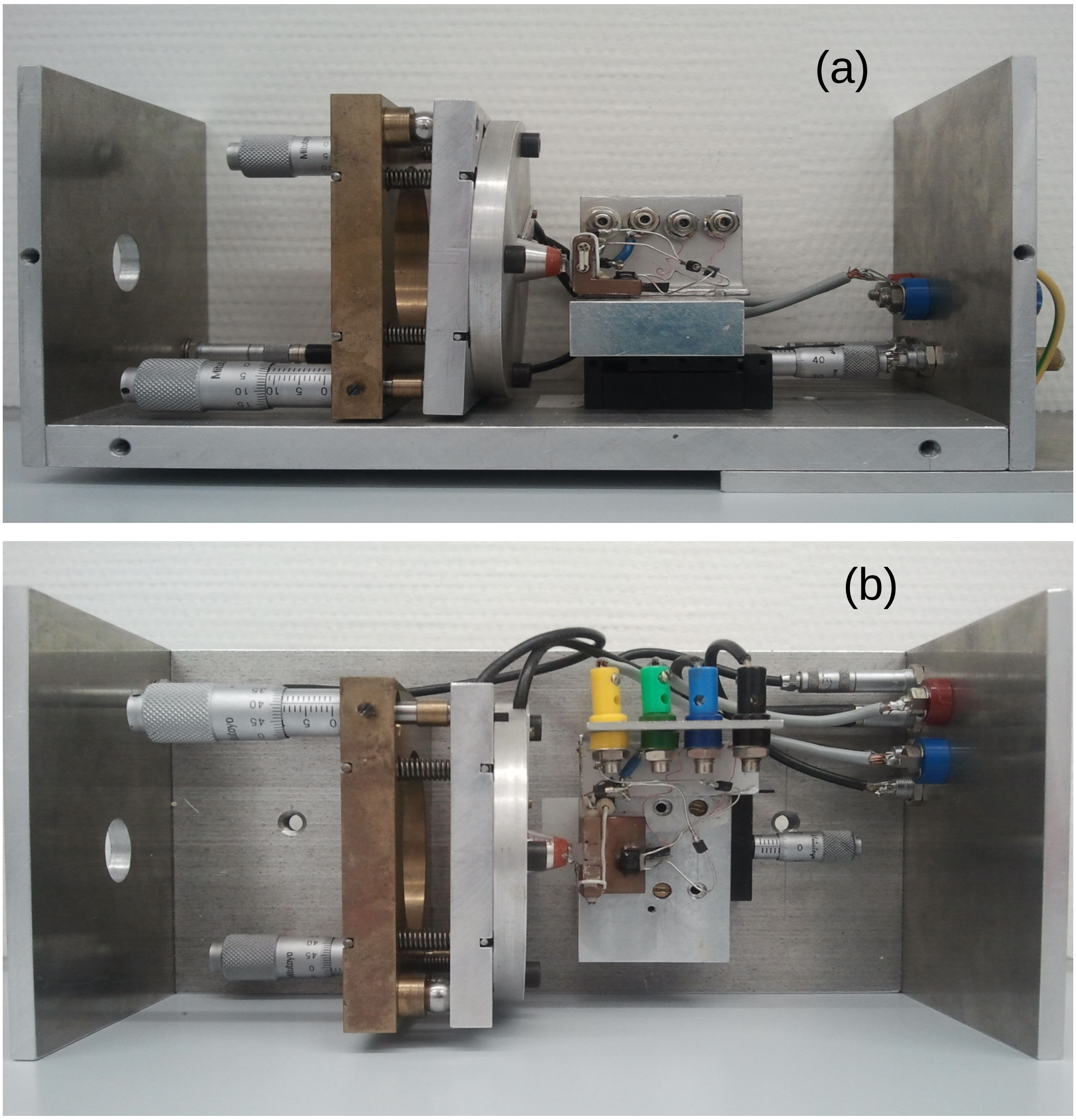

Figure S2. Kelvin probe device in (a) side view and (b) top view..

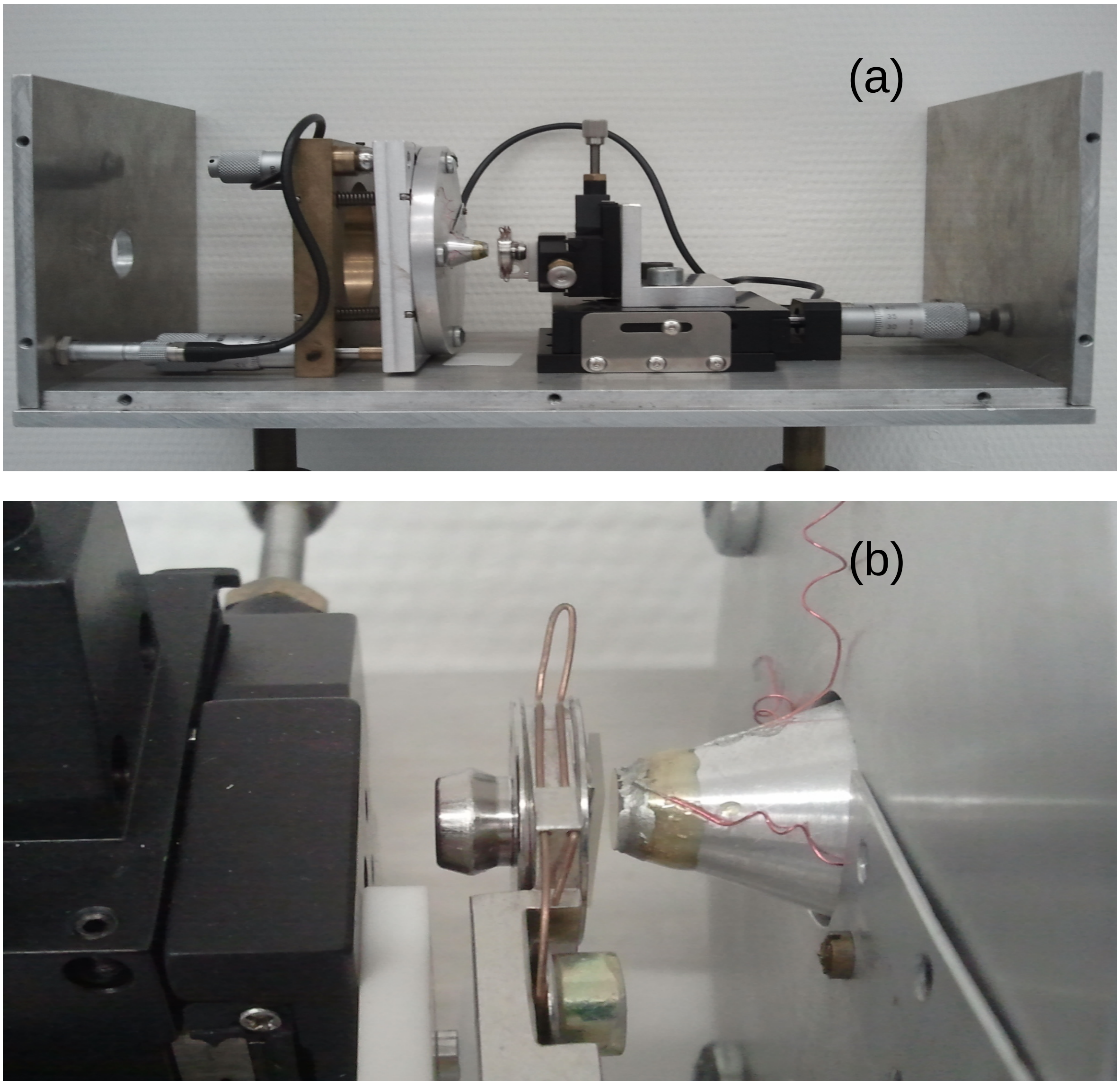

Figure S3. (a) Modified Kelvin probe device. (b) Zoom picture of sample and probe.

3. Reference surface photovoltage data on material systems different from $SrTiO_3$

The performance of the Kelvin probe device was cross-checked with two samples of a conventional semiconductor: n- and p-type silicon, for which SPV data are available in the literature. Figure S4 comparatively shows the behavior of the contact potential upon super-bandgap illumination for both Si samples. While the temporal response is too fast to be resolved with a Kelvin probe detection scheme, both the sign and the relative magnitudes (much smaller SPV in p- than in n-type Si) of the SPV are in accordance with the literature [5]. The signal-to-noise ratio is in the same range as in the measurements on highly resistive oxides.

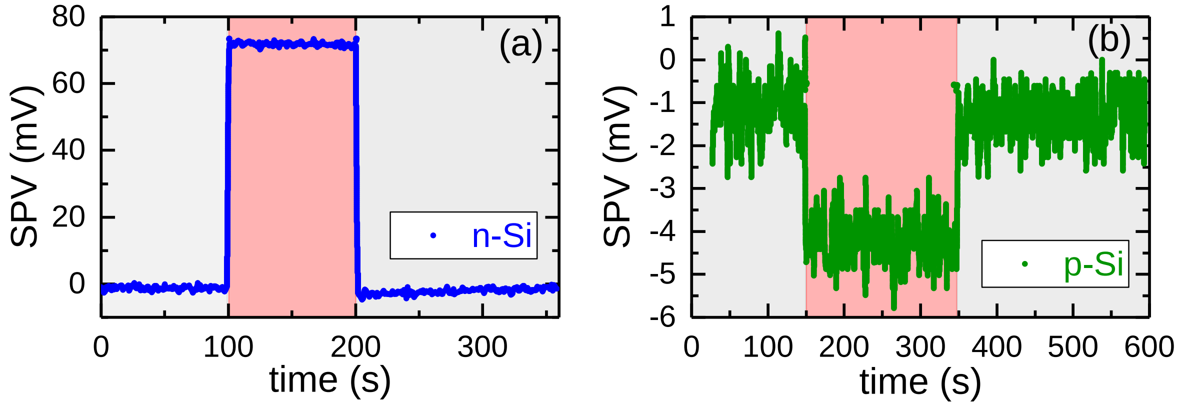

Figure S4. Surface photovoltage in (a) n-type and (b) p-type silicon upon superbandgap illumination (wavelength: 780 nm, intensity: 0.5 mW, spot size = probe area). Darkness periods are indicated by grey color and illumination periods by red color, respectively.

For a detailed discussion of SPV data, acquired with the described Kelvin probe setup on a different oxide system, namely the currently intensively investigated $LaAlO_3/SrTiO_3$ heterosystem, refer to our in-depth study [6].

Bibliography

1. Abramowitz, M.; Stegun, I. *Handbook of Mathematical Functions, With Formulas, Graphs, and Mathematical Tables*; Dover Publications Inc.: New York, 1970.
2. Weisstein, E.W. Catalan's Triangle. In "*MathWorld* - A Wolfram Web Resource." http://mathworld.wolfram.com/CatalansTriangle.html. [Online; accessed 08-October-2018].
3. Sloane, N.J.A. Sequence A009766. In "The On-Line Encyclopedia of Integer Sequences", http://oeis.org/A009766. [Online; accessed 08-October-2018].
4. Weisstein, E.W. Hypergeometric Function. In "*MathWorld* - A Wolfram Web Resource." http://mathworld.wolfram.com/HypergeometricFunction.html. [Online; accessed 08-October-2018].
5. Kuhlmann, W.; Henzler, M. Non-equilibrium surface state properties at clean cleaved silicon surface as measured by surface photovoltage. *Surf. Sci.* **1980**, *99*, 45–58. doi:10.1016/0039-6028(80)90575-0.
6. Beyreuther, E.; Paparo, D.; Thiessen, A.; Grafström, S.; Eng, L.M. Conducting and insulating $LaAlO_3/SrTiO_3$ interfaces: A comparative surface photovoltage investigation. *J. Appl. Phys.* **2013**, *114*, 243709. doi:10.1063/1.4858376.
